# A New CNN-Based Single-Ingredient Classification Model and Its Application in Food Image Segmentation

**DOI:** 10.3390/jimaging9100205

**Published:** 2023-09-29

**Authors:** Ziyi Zhu, Ying Dai

**Affiliations:** Faculty of Software and Information Science, Iwate Prefectural University, Takizawa, Iwate 020-0693, Japan; g236t002@s.iwate-pu.ac.jp

**Keywords:** CNN architecture, single-ingredient classification model, food ingredient segmentation, evaluation metrics, hierarchical multi-level learning

## Abstract

It is important for food recognition to separate each ingredient within a food image at the pixel level. Most existing research has trained a segmentation network on datasets with pixel-level annotations to achieve food ingredient segmentation. However, preparing such datasets is exceedingly hard and time-consuming. In this paper, we propose a new framework for ingredient segmentation utilizing feature maps of the CNN-based Single-Ingredient Classification Model that is trained on the dataset with image-level annotation. To train this model, we first introduce a standardized biological-based hierarchical ingredient structure and construct a single-ingredient image dataset based on this structure. Then, we build a single-ingredient classification model on this dataset as the backbone of the proposed framework. In this framework, we extract feature maps from the single-ingredient classification model and propose two methods for processing these feature maps for segmenting ingredients in the food images. We introduce five evaluation metrics (IoU, Dice, Purity, Entirety, and Loss of GTs) to assess the performance of ingredient segmentation in terms of ingredient classification. Extensive experiments demonstrate the effectiveness of the proposed method, achieving a mIoU of 0.65, mDice of 0.77, mPurity of 0.83, mEntirety of 0.80, and mLoGTs of 0.06 for the optimal model on the FoodSeg103 dataset. We believe that our approach lays the foundation for subsequent ingredient recognition.

## 1. Introduction

With the rapid development of deep learning techniques in recent years, food computing [1] has emerged as an interesting field owing to its wide range of applications in health, culture, and other domains. It is important to analyze and understand food images from different perspectives. For the healthcare domain, food computing can help to understand food images from different perspectives, such as nutrition estimation, food choices, and healthy eating recommendations. For the food retailer domain, restaurants and food retailers can utilize food computing technology to automate order processing, manage inventory, or offer personalized dietary recommendations to customers.

Among various tasks in food computing, food recognition has attracted considerable research interest in recent years. Existing studies include deep-based recognition, which leverages different deep-food recognition models [2,3,4,5,6]. However, we have observed that these research studies focus on identifying food by the name of the dish. There are two key limitations to this approach: (1) there are limitations for nutritional analysis, as dishes with the same name can contain different ingredients; (2) the construction of a dish name classification model is impractical, because the dish names are endlessly variable.

Given these limitations, some studies [7,8,9] have focused on food ingredient recognition, because ingredient categories are limited and usually defined according to standard food taxonomy.

Food ingredient recognition involves the automatic identification of multiple ingredients in a food image. This improves the accuracy of dietary assessment, food tracking, and nutritional analysis. The accurate identification of all meal ingredients is crucial for these applications.

However, food ingredient recognition poses challenges due to the high variability in ingredient appearance as well as visual similarities among some different ingredients. For instance, the appearance of an egg can vary significantly with different cooking methods. Some ingredients, such as spinach and Bok choy, exhibit visual similarities in color, texture, and shape. These factors collectively contribute to the complexity of ingredient classification.

Food ingredient recognition, as studied in [10,11,12,13,14], is commonly regarded as a multi-label classification task. To enhance performance, some approaches [12,13] based on multi-task and region-based deep learning are proposed. Furthermore, Chen et al. [14] deployed a multi-relational graph convolutional network that considered the relationships between different ingredients, including ingredient hierarchy, co-occurrence, and cooking and cutting methods. However, these previous works still have some limitations: (1) Existing models are based on multi-label ingredient recognition. However, this method is not optimal because it does not directly and accurately learn visual ingredient representations, and is often influenced by the mutual interference of adjacent ingredients in the images during training and testing. (2) There are no standardized ingredient datasets which cover a wide range of ingredient categories.

To exclude the inference of adjacent ingredients in ingredient recognition, some studies [15,16,17] explored methods for food ingredient segmentation. These studies trained segmentation networks on pixel-level annotated ingredient datasets, such as FoodSeg103 [15]. However, pixel-level annotation for each image is time-consuming.

A database called AI4Food-NutritionDB is developed in [18]. This database categorizes foods based on a nutritional four-level pyramid structure and analyzes food recognition tasks using a nutrition taxonomy. However, this database does not account for biological inherent hierarchical structure among the ingredients.

To address these challenges, in this paper, we propose a new framework for ingredient segmentation utilizing feature maps of the CNN-based Single-Ingredient Classification Model that is trained on the dataset with image-level annotation. To train this model, we first introduce a standardized biological-based hierarchical ingredient structure and construct a single-ingredient image dataset based on this structure. Then, we build a single-ingredient classification model on this dataset as the backbone of the proposed framework. In this framework, we extract feature maps from the single-ingredient classification model and propose two methods to process these feature maps for segmenting ingredients in the food images. We introduce five evaluation metrics (IoU, Dice, Purity, Entirety, and Loss of GTs) to assess the performance of ingredient segmentation in terms of ingredient classification.

Our main contributions are:

1. A single-ingredient image dataset was constructed based on food taxonomy standards [19,20] to train the CNN-based single-ingredient classification model. This dataset covers a wide range of ingredient categories, and contains various individual ingredient images with various cutting and cooking methods.
2. Some single-ingredient classification models with different architectures were trained on the above dataset, so as to obtain an optimal model utilized to ingredient segmentation.
3. A new multi-ingredient segmentation framework utilizing the above model as an extractor of feature maps was proposed. Furthermore, two methods were introduced for processing the feature maps to generate ingredient masks for the ingredient segmentation.

This paper is organized follows. In Section 2, we provide a review of relevant works. In Section 3, we introduce a new individual ingredient image dataset. In Section 4, we introduce a novel CNN-based architecture for the single-ingredient classification model. In Section 5, we present a new multi-ingredient segmentation framework that utilizes the aforementioned model. Section 6 covers the introduction of five metrics for evaluating ingredient segmentation. In Section 7, we analyze the performance of the single-ingredient classification model and the proposed multiple-ingredient segmentation framework. Finally, in Section 8, we present our conclusions.

## 2. Related Work

In this section, we briefly review several related studies including food ingredient segmentation, multi-task learning, and K-Means Clustering.

### 2.1. Food Ingredient Segmentation

Before discussing food ingredient segmentation, we briefly introduce food segmentation. Food segmentation aims to segment each food category and its pixel-wise location within a food image. Aguilar et al. [21] combined food/non-food binary masks and food localization bounding boxes to achieve food segmentation. Sharma et al. [22] introduced a network called GourmetNet, which adopts the Waterfall Atrous Spatial Pooling (WASPv2) module, and employs dual attention (channel and spatial) mechanisms for multi-scale waterfall features to improve the food segmentation performance. Okamoto et al. [23] introduced a region-based segmentation model for multiple-dish segmentation. Liang et al. [24] proposed a ChineseFoodSeg approach, which uses the color and texture features of super pixels to separate different dishes from a single plate.

Food ingredient segmentation has recently emerged as a promising means of identifying each ingredient category and its specific location within a food image at the pixel level. However, ingredient segmentation poses notable challenges due to the inherent high variability in ingredients. For instance, eggs exhibit significant intra-class variance depending on the cooking method employed, such as boiling or steaming. On the other hand, certain categories, such as spinach and kale, present a high inter-class similarity as they are both green leaves and are often prepared in recipes of similar shapes and sizes. One additional challenge in ingredient segmentation is the similarity between certain ingredients and the background. Wu et al. [22] proposed a food image segmentation framework, which consists of two modules: Recipe Learning Module (ReLeM) and Image Segmentation module. Specially, ReLeM incorporates recipe information and integrates recipe embedding with the visual representation of a food image to enhance the visual representation of an ingredient. Wang et at. [23] proposed a Swin Transformer-based pyramid network to combine multi-scale features from the global and local regions of the food image for food image segmentation. Xia et al. [24] proposed a network consisting of two subnetworks to refine the boundaries of the ingredient segmentation. Specifically, this study incorporates both Hyperspectral Imaging (HSI) and RGB images as inputs for the feature extraction. The latest study, Segment Anything [25], introduced an efficient transformer-based model that unifies various segmentation tasks into a general framework to implement class-agnostic instance segmentation.

However, to the best of our knowledge, all the aforementioned studies rely on training proposed segmentation models on training datasets with pixel-level annotations. Acquiring such datasets, especially for food ingredient segmentation, requires a significant amount of manual labeling, which is time-consuming and prone to errors. In contrast to these methods, we propose a weakly supervised segmentation approach that requires only single-ingredient images and their corresponding labels, thereby reducing the need for pixel-level annotation.

### 2.2. Food-Related Public Datasets

Along with research on food ingredient recognition, there are some large-scale datasets, such as VIREO Food-172 [26] and ISIA Food 500 [27]. VIREO Food-172 is one of the first datasets to consider these ingredients. It contains 110,241 images from 172 food categories. The images were manually annotated based on 353 ingredients.

Several public datasets are available for food segmentation. Food-201 [28] contains 12,093 images with 201 food categories and 29,000 dishes. UECFoodPix COMPLETE [29] is another widely used food image dataset that contains 10,000 food images across 102 food categories and 14,011 masks. However, these datasets only provide dish-level and not ingredient-level segmentation masks. FoodSeg-103, proposed in [15], consists of 7118 images and more than 40,000 masks, covering 103 food ingredient categories. FoodSeg-103 is the first large-scale food image dataset with ingredient-level segmentation masks. HSIFoodIngr-64, proposed in [17], contains 21 dish and 64 ingredient categories. This study provides annotated HSI images that contain more informative properties of ingredients.

In this study, we use the FoodSeg103 dataset to evaluate the performance of the proposed method for ingredient segmentation, and use the UECFoodPix COMPLETE dataset to evaluate the performance of food segmentation. As the dataset from study [24] has not yet been made publicly available, we did not compare our results with it.

### 2.3. Multi-Task Learning

Multi-task learning is widely used approach in computer vision and plays a critical role in image classification [30,31], object detection [32], and semantic segmentation [33,34]. Li et al. [35] proposed a multi-task network cascade network that consists of three stages for each task, and a sequential feature-sharing method among tasks. Another study [36] introduced a hierarchical network via setting the supervision of low-level tasks in the bottom layers and high-level tasks in the top layer of the model.

Our study was inspired by the bottom-up supervisor-setting method of study [36]. We introduced a multi-level learning strategy for single-ingredient classification.

### 2.4. K-Means Clustering

Clustering is a simple and effective method of image segmentation. Specifically, k-means clustering is the most widely used method, found in many works [37,38]. The main concept of image segmentation using the k-means method is to partition a collection of pixels in an image into k clusters, based on their similarity. Zheng et al. [38] introduced an adaptive k-means algorithm for LAB color space to improve the performance of image segmentation. Caron et al. [39] proposed a deep neural network called deep embedded clustering (DEC), which incorporates both an autoencoder and a clustering module. Van et al. [40] introduced a method for clustering learned pixel embeddings into groups to address unsupervised segmentation.

## 3. Dataset

In this section, we will first introduce the hierarchical ingredient structure based on food taxonomy standards [19,20]. Then, we will introduce the single-ingredient image dataset for training the single-ingredient classification model.

### 3.1. Hierarchical Ingredient Structure

**Figure 1:**
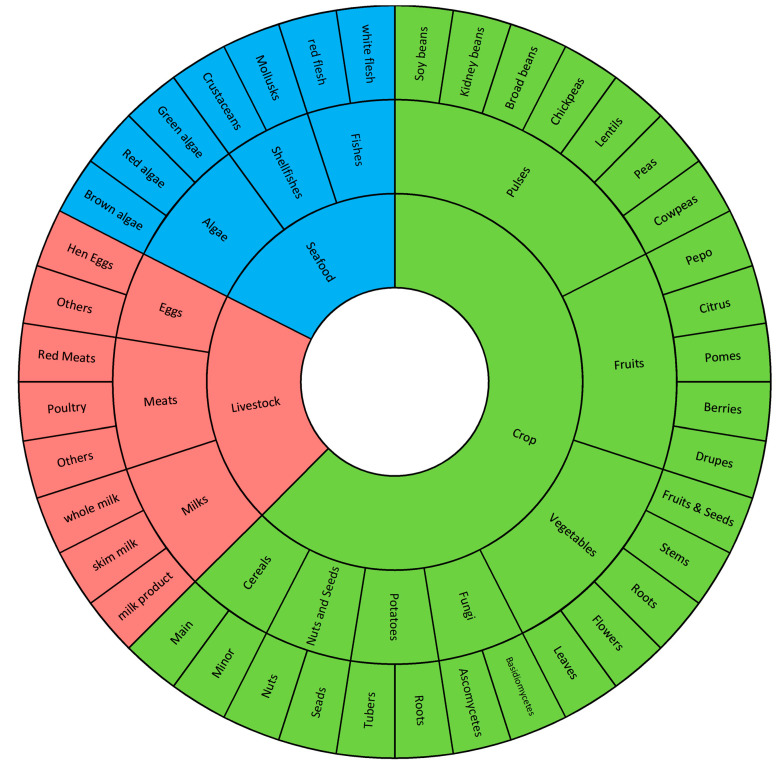
Architecture of the first three levels of the hierarchical ingredient structure.

In a previous study [41], we proposed a three-level structure for ingredient categories based on the Japanese food taxonomy ([19,20]; see Figure 1). This structure is biological, and now we expand it by adding level 4 ingredient categories based on the same taxonomy as in [20]. As a result, we have a four-level hierarchical structure for the ingredient categories.

In this four-level hierarchical structure, level 1 ingredient categories are defined based on the standard described in [16], including Crop, Livestock, and Seafood. Level 2 to level 4 ingredient categories are defined based on another standard described in [20]. Each ingredient category at a lower level belongs to only one type of ingredient at a higher level. For example, “Fruits”, “Vegetables”, and “Meats” are level 2 ingredient categories, “Fruits” and “Vegetables” belong to “Crop” (level 1 ingredient category), while “Meats” belongs to “Livestock”, as shown in Figure 1. As a result, level 2 includes 13 ingredient categories, level 3 includes 32 ingredient categories, and level 4 includes 110 commonly used ingredient categories, providing a comprehensive coverage of food taxonomy.

### 3.2. Single-Ingredient Image Dataset (SI110)

**Figure 2:**
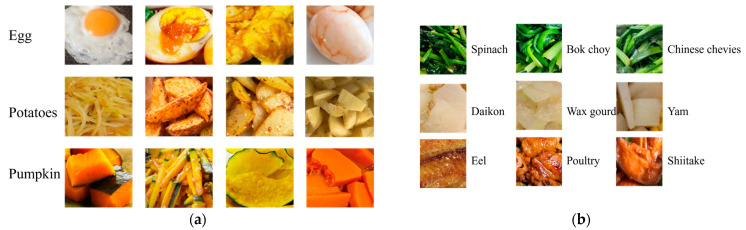
Intra-class variance and inter-class similarity. (**a**) Samples of three ingredients with high intra-class variance. (**b**) Samples of three sets exhibiting high inter-class similarity.

In this work, we construct a novel single-ingredient image dataset. In order to solve the problem of high intra-variance of ingredients in the food images, we collect individual ingredient images with various cutting and cooking methods. We follow several criteria for data collection. First, we exclude invisible ingredients such as salt and sugar because our goal is to recognize visually observable ingredients in food images. Second, we ensure that each single-ingredient image contains only one type of ingredient as defined in the level 4 category list. This is achieved through capturing single-ingredient images or extracting single-ingredient regions from the food images manually. Third, based on the different cooking conditions, we collect as many visual variants as possible for each ingredient category. For example, we gather different visual appearances of eggs, potatoes, and pumpkins under various cooking conditions (Figure 2). Finally, we ensure that five to ten images are selected for each type of visual appearance of each ingredient category to prevent training bias towards a specific visual appearance.

**Figure 3:**
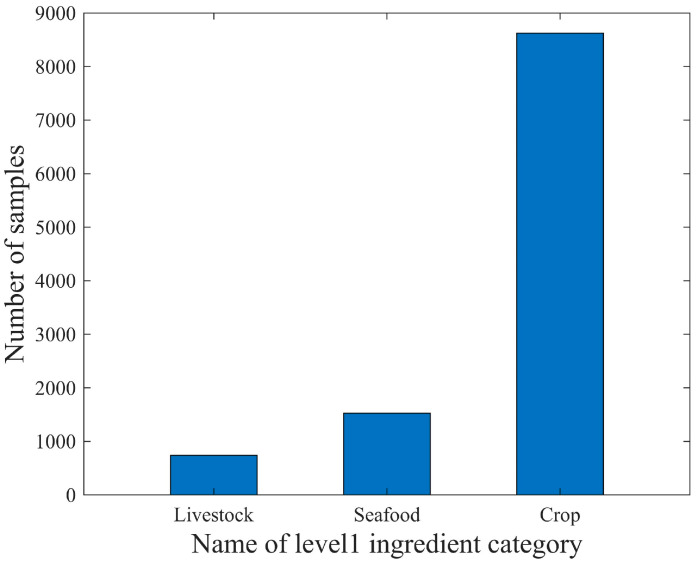
The distribution of sample counts for ingredient categories in level 1 of the SI110 dataset.

**Figure 4:**
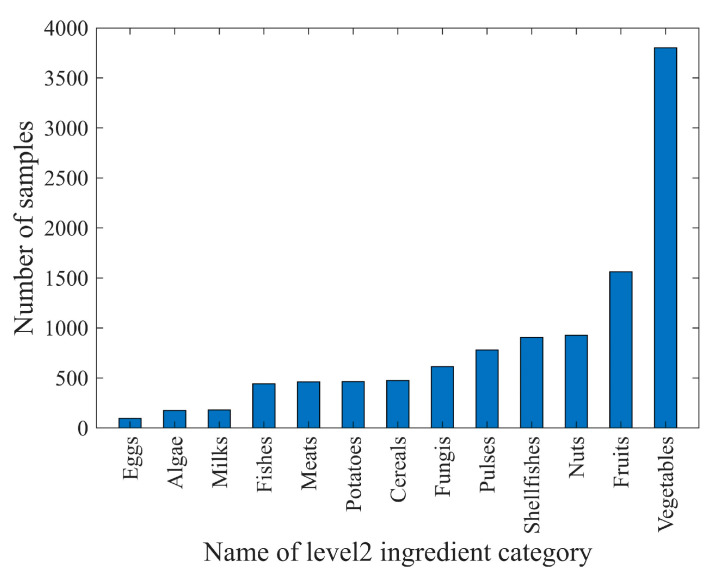
The distribution of sample counts for ingredient categories in level 2 of the SI110 dataset.

**Figure 5:**
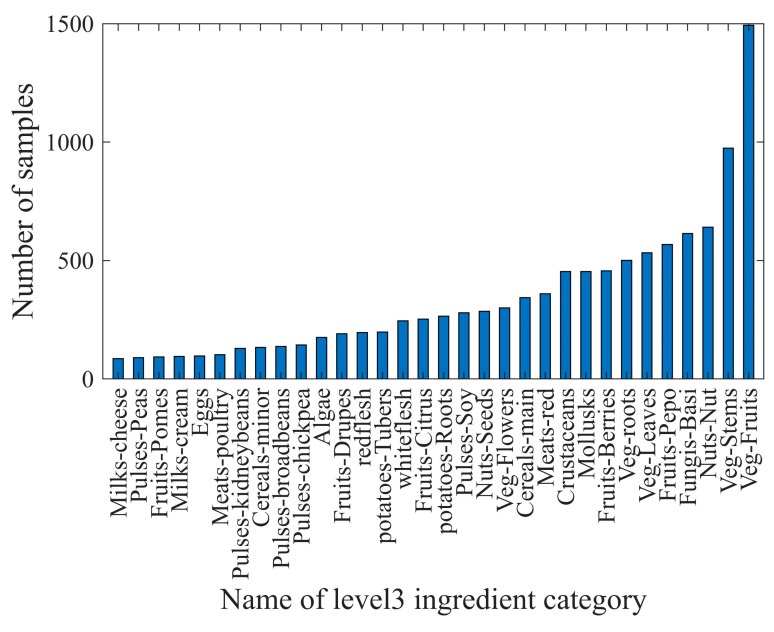
The distribution of sample counts for ingredient categories in level 3 of the SI110 dataset.

**Figure 6:**
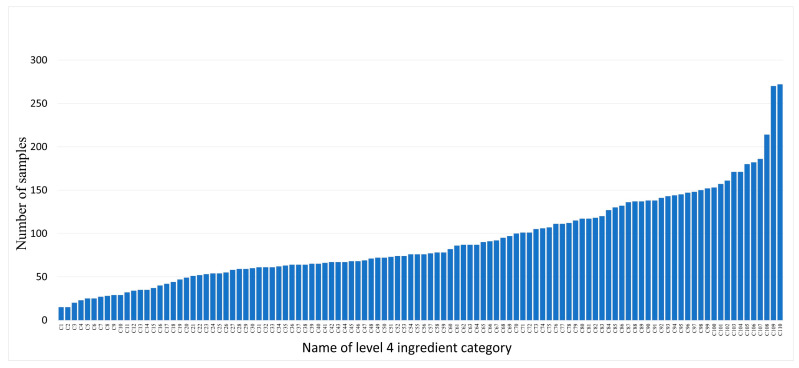
The distribution of sample counts for ingredient categories in level 4 of the SI110 dataset.

In the scope of the 110 food ingredient categories, we collect food images from Google Pictures using English, Chinese, and Japanese keywords including these ingredients. Subsequently, we perform several rounds of processing on the collected images, including: (1) Cropping out the regions that have the individual ingredient. These cropped regions were then used as single-ingredient samples. (2) Ensuring that each food ingredient has five to ten samples for each visual variation. Currently, SI110 contains 10,750 single-ingredient images, including 110 level 4 ingredient categories, covering the entire range of food taxonomy. The images are then assigned to the corresponding categories at the three upper levels. The distribution of ingredient categories for each level is shown in Figure 3, Figure 4, Figure 5 and Figure 6. The detailed names corresponding to the notations of the horizontal coordinates in Figure 6 are shown in Appendix A.

Data samples at each level follow a long-tailed distribution. For example, at level 4, ingredient categories that offer many different cooking ways, such as shrimp and wheat products, contain more than 250 samples. However, ingredients that are not prepared in many different ways, such as green caviar and raspberry, contain only about 20 samples.

The SI110 dataset is randomly divided into 80% for training and 20% for testing the single-ingredient classification model.

## 4. Single-Ingredient Classification Model

In this section, we propose a new CNN-based architecture for a single-ingredient classification model trained on the SI110 dataset.

### 4.1. Proposed CNN-Based Architecture

**Figure 7:**
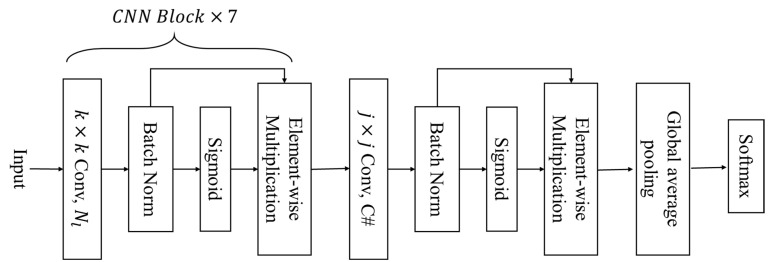
Architecture of the CNN-based AttNet network, where k and j are the kernel size of the convolutional layer, $N_{l}$ represents the channel number of the convolutional layer in the CNN block l, and C# indicates the number of categories.

In this subsection, we present AttNet, a novel CNN-based architecture for single-ingredient classification. The complete structure of an AttNet is shown in Figure 7. Inspired by EfficientNet [42], we include a sigmoid layer followed by an element-wise multiplication layer in each of CNN Blocks. AttNet consists of eight CNN blocks with identical structures. Each CNN block has four layers. The first layer is the convolutional layer followed by a batch normalization layer. We then follow a sigmoid layer to compute the activation value of the feature map from the batch normalization layer. Its principle is to map the input feature values to a probability range between 0 and 1. Finally, we add an element-wise multiplication layer to multiply the activation value with the feature map output from the batch normalization layer. Finally, we add a global average pooling layer after the last CNN block and add a classification layer at the end. We present the whole network parameter in Table 1. 

In this work, we explore the use of two different kernel sizes for the convolutional layer in the CNN blocks. Firstly, we use a kernel size of 1. We refer to this network as the AttNet (1). We chose to only use 1 × 1 convolutional layer, because it significantly reduces computational cost and memory size. Secondly, we use a kernel size of 3, which is commonly utilized in CNN networks. We refer to this network as the AttNet (3). Furthermore, we propose two variants of AttNet, which are: (1) AttNet (1 + 3), where the first seven CNN blocks use 1 × 1 convolutional layers and the last CNN block uses a 3 × 3 convolutional layer; (2) AttNet (3 + 1), where the first seven CNN blocks use 3 × 3 convolutional layers and the last CNN block uses a 1 × 1 convolutional layer.

Moreover, we fine-tune ResNet18 [43] and EfficientNetB0 [42] for single-ingredient classification, because they are both popular and widely used architectures for image processing and segmentation tasks. We modify both the pre-trained models by replacing their last convolutional layer with a 1 × 1 convolutional layer with C# channels, followed by a global average pooling layer, and finally adding a classification layer.

Finally, all models, including AttNets, the modified ResNet18, and EfficientNet-B0, are trained on the SI110 train dataset.

### 4.2. Training Models

**Figure 8:**
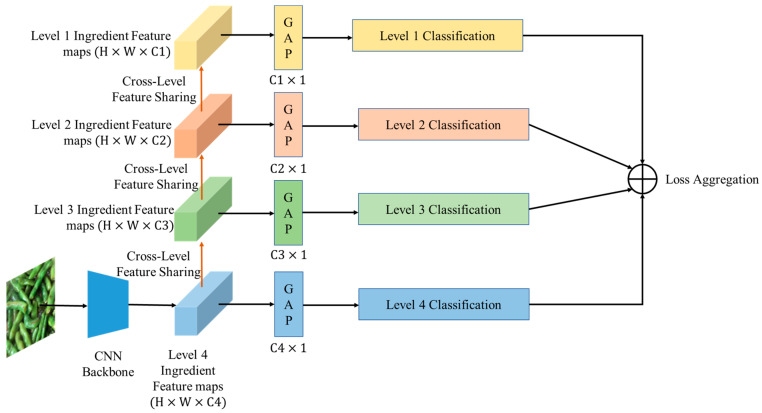
The diagram of multi-level learning of multi-level single-ingredient classification that utilizes a bottom-up feature-sharing mechanism to facilitate multi-level learning, where {C1, C2, C3, C4} indicates the number of categories at each level.

**Figure 9:**
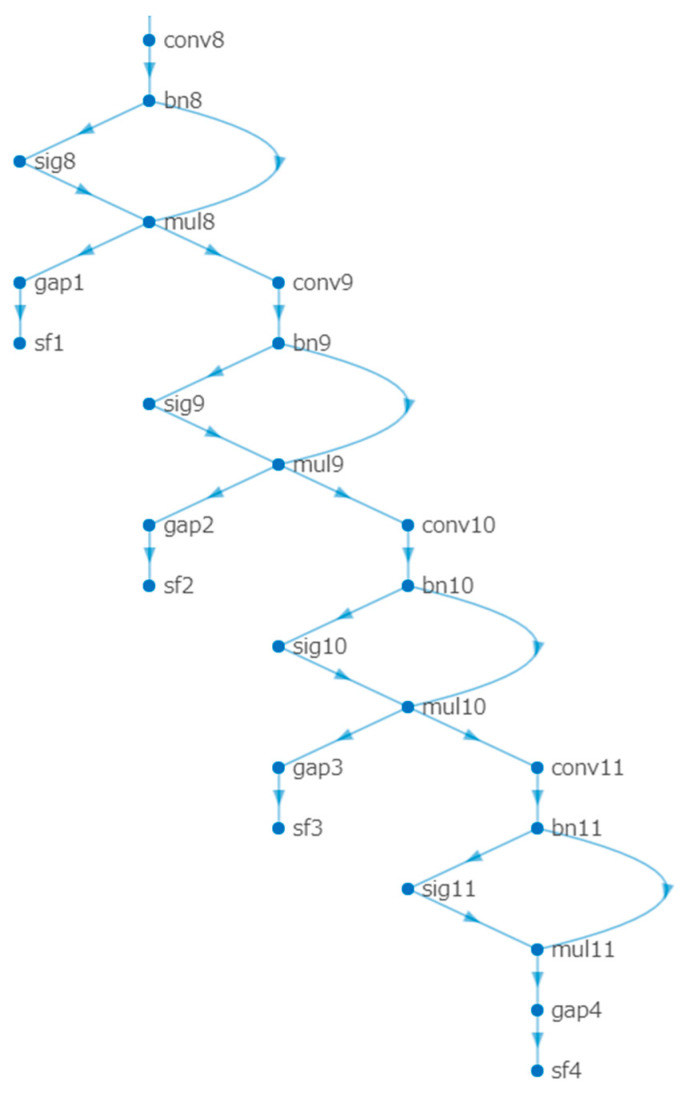
The diagram of multi-level feature-sharing mechanism with a bottom-up feature-sharing mechanism. CNN Blocks are sequentially stacked from level 4 to level 1 (conv8 to conv11), where the gap represents the global average pooling layer and sf refers to the softmax function.

In this subsection, we introduce a method for training a single-ingredient classification model using the SI110 dataset. First, we present the baseline work, which is a single-level learning method that trains only the classification model for level 4 ingredient categories. The models trained using this method are referred to as SLMs (Single-Level Models). Furthermore, we propose a multi-level learning strategy for single-ingredient classification that simultaneously leverages four levels of ingredient information from the hierarchical structure. A diagram of the proposed method is shown in Figure 8. We employ a bottom-up feature-sharing mechanism via setting an individual CNN Block for each level after the seventh CNN Block. These CNN Blocks are sequentially stacked from level 4 to level 1. Additionally, for each level’s CNN Block, we include a global average pooling layer (gap), followed by a Softmax layer to perform classification for each level. We demonstrate the bottom-up feature-sharing mechanism in Figure 9.

The models trained using this multi-level learning method are referred to as MLMs (Multiple-Level Models). During the training process, we compute the standard cross-entropy loss for each level′s ingredient classification, and optimize the weighted sum of the four losses with different weights. To ensure balanced training, we assign a higher weight to bottom levels than upper levels. This is because the number of bottom ingredient categories is greater than the number of upper ingredient categories, and we aim to account for this discrepancy in the training process. We define the total loss function for multi-level ingredient classification as follows:

(1) $$L_{total}=\sum_{i}^{4}\lambda_{i}L_{i}(sf(z^{i},y^{i})$$

where $L_{i}$ means the cross-entropy loss function for level i, sf means the Softmax function, $z^{i}$ represents the output of the global average pooling layer, and $y^{i}$ represents the ground-truth class label for level i. In this work, we adopt a fixed set of weights {1.0, 0.5, 0.3, and 0.1} in a descending order from level 4 to level 1. The purpose of assigning decreasing weights to classification tasks at different levels is to emphasize the importance of level 4 ingredients during the training process. We decrease the weights of tasks at upper levels. Based on the hierarchical structure, we gradually decrease the importance of upper-level tasks via considering the cross-level distance between different levels and the fourth level.

## 5. Ingredient Segmentation Framework

**Figure 10:**
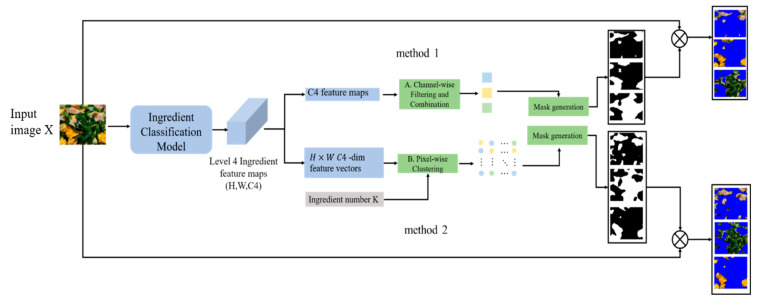
The diagram multiple ingredient segmentation framework.

Based on the above single-ingredient classification model, we propose a new framework for ingredient segmentation, as shown in Figure 10. The framework extract feature maps of multiple-ingredient food images using a pre-trained single-ingredient classification model. The advantage of this framework is that it does not need to construct the pixel-level annotated dataset for the segmentation, which are typically required by existing ingredient segmentation networks. This offers a more practical and cost-effective solution for food ingredient segmentation. Preparing pixel-level annotated datasets is particularly challenging and time-consuming, largely due to issues such as fuzzy boundaries and overlapping of food ingredients. 

The input image is denoted by X. Feature maps are extracted from the last convolutional layer of the model and are denoted by $f\left(X\right)$, where $f\left(X\right)\in R^{H\times W\times C4}$ and C4 is the number of level 4 ingredient categories. Subsequently, the feature maps $f\left(X\right)$ are processed to generate the ingredient masks. In the following section, we introduce two methods for feature-map processing.

In Method 1, we first transform the feature maps $f\left(X\right)$ into 2D feature maps with C4 channels. The first step is to filter the C4 feature maps. We calculate the global average value of each feature map, then normalize it using the sigmoid function to compute a score. Then, we selected the feature maps with scores greater than the threshold of 0.5. The second step is to combine the feature maps. We calculate the correlation coefficient for each pair of filtered feature maps. When the correlation coefficient exceeds the threshold of 0, a pair of feature maps is merged into one feature map. The reasoning behind this step is that the positively correlated feature maps tend to encode redundant information. By merging these results, we obtain a more complete activation result for a particular component. Finally, we binarize all processed feature maps to create masks for ingredient segmentation.

In Method 2, we transform the feature maps into H × W pixel-wise feature vectors with C4-dimensionals. We then apply k-means clustering to these vectors to obtain K clusters, where K represents the number of ingredients in the dish image and we assume that the value K is known in advance in this paper. Each cluster, which is composed of pixels, generates a mask for the ingredient segmentation.

Finally, we resize the masks to the same size as the input image and then apply element-wise multiplication to each mask and input image to obtain the segments of the ingredients in the food image.

## 6. Segmentation Evaluation Metrics

In this section, we introduce five metrics to evaluate the performance of ingredient segmentation. IoU is a measurement of the overlap between the predicted segmentation mask and the ground truth mask. It is calculated by dividing the intersection of the predicted and ground truth regions by their union. The Dice coefficient is another metric which also quantifies the similarity between the predicted and ground truth masks. It is calculated by taking twice the intersection area of the predicted and ground truth masks and dividing it by the sum of the areas of the predicted mask and ground truth mask. In this study, we employ these metrics to evaluate the performance of food ingredient segmentation.

In addition to IoU and Dice metrics, the purity and entirety of segmentation are crucial aspects for recognizing ingredients in the next step. Furthermore, the region loss of the ingredient of the ground truth (LoGT) should also be considered for the evaluation because the loss of the ingredient makes it impossible to be recognized. The definitions of Purity and Entirety are equivalent to those proposed in [44]. Specifically, Purity measures the ratio of the ingredient of the ground truth (GT) contained in a segment, and Entirety measures the ratio of the segment contained in an ingredient of the GT.

All the mentioned metrics are calculated using Equations (2)–(6), and it should be noted that IoU is a comprehensive metric of Purity and Entirety:

(2) $$intersection\;over\;union(IoU)=\frac{\left|A\cap B\right|}{\left|A\cup B\right|}$$

(3) $$Dice=\frac{2\times \left|A\cap B\right|}{\left|A|+|B\right|}$$

(4) $$purity=\frac{\left|A\cap B\right|}{\left|B\right|}$$

(5) $$entirety=\frac{\left|A\cap B\right|}{\left|A\right|}$$

(6) $$LoGTs=\frac{\left|{⋃}_{i}^{I}A_{i}\right|-\left|{⋃}_{i}^{I}A_{i}\cap {⋃}_{j}^{J}B_{j}\right|}{\left|{⋃}_{i}^{I}A_{i}\right|}$$

where A and B denote the masks of the GT and the segment, respectively, I denotes the number of GTs in the sample, and J is the number of segments in the sample.

Moreover, we calculate the mean IoU (mIoU), mean Dice (mDice), and mean purity (mPurity) by averaging the maximum purity of all segments, and the mean entirety (mEntirety) by averaging the maximum entirety of all GTs. Mean LoGTs (mLoGTs) are calculated by computing the average region loss of the foreground for each image.

## 7. Experiments and Analysis

To evaluate the generalization of the proposed method, we conduct assessments on both the FoodSeg103 dataset, which is a publicly available dataset specifically designed for ingredient segmentation, and the UEC-FoodPix Complete dataset, which is the most recognized dataset for food segmentation. There are other food databases, such as UNIMIB2016. Because our primary objective in this work is ingredient segmentation, we prioritized the evaluation using the UEC-FoodPix Complete dataset. We will extend the evaluation of the generalization on other public datasets in our next work.

In this section, we evaluate: (1) the performance of the single-ingredient classification model in single-ingredient identification; (2) the performance of the ingredient segmentation framework for multi-ingredient segmentation; (3) the performance of the ingredient segmentation framework for food segmentation.

### 7.1. Implementation Setups

The experiments were implemented on a computer with the following specifications: an Intel(R) Core i7-10870H CPU @ 2.20 GHz and an NVIDIA GeForce RTX 3060 Laptop GPU, with 16 GB memory. The operating system used was Windows 11, and the codes were written in MATLAB (2022a). In the training process, we trained all single-level single-ingredient classification models and all multi-level single-ingredient classification models on the SI110 training dataset. The single-level models were trained for 30 epochs, while the multi-level models were trained for 50 epochs. We used a mini-batch size of 32 and an initial learning rate of 3e-2. To facilitate learning, we implemented a piece learning rate schedule, where the learning rate was multiplied by 0.2 when decreased. Furthermore, we utilized the Adam optimizer with a squared gradient decay factor of 0.9.

To assess the effectiveness of our proposed method, we conducted the evaluations in two stages. Firstly, we evaluated the performance of single-ingredient classification on the SI110 test dataset. SI110 contains 2150 test images with single ingredients in a food image. Next, we evaluated the performance of ingredient segmentation on the FoodSeg103 dataset. FoodSeg103 contains 2135 test images with multiple ingredients in a food image with pixel-level annotations.

### 7.2. Evaluation on the Single-Ingredient Classification Model

In this section, we present a thorough evaluation of our proposed AttNets and two pretrained models, ResNet18 and EfficientNet-B0, for single-ingredient identification. To explore the effectiveness of different kernel sizes in the CNN blocks of AttNets, we designate four types of AttNets, all with the same architecture, but varying kernel sizes:

1. AttNet (1): uses a convolutional layer with kernel size = 1 in each CNN block;
2. AttNet (1 + 3): uses a convolutional layer with kernel size = 1 in each CNN block, except for the convolutional layer with kernel size = 3 in the last CNN block;
3. AttNet (3 + 1): uses a convolutional layer with kernel size = 3 in each CNN block, except for the convolutional layer with kernel size = 1 in the last CNN block;
4. AttNet (3): uses convolutional layer with kernel size = 3 in each CNN block.

We employ four metrics to evaluate the performance of single-ingredient classification: accuracy, precision, recall, and F1-score. The experimental results are presented in Table 2.

In terms of the performance of SLM, our observations reveal that the modified ResNet18 achieves the highest performance among the SLMs. It attains an accuracy of 0.8684, a precision of 0.8498, recall of 0.8571, and an F1 score of 0.8466. Among the AttNet models, AttNet (1) demonstrates the top performance, with an accuracy of 0.2712, a precision of 0.1933, recall of 0.1955, and an F1 score of 0.1693.

Regarding the performance of MLM, we found that the modified EfficientNet-B0 exhibits the best performance, with an accuracy of 0.8684, a precision of 0.8307, recall of 0.8253, and an F1 score of 0.8168. Among the AttNet models, AttNet (3 + 1) achieves the highest performance, with an accuracy of 0.387, a precision of 0.41, recall of 0.3254, and an F1 score of 0.324.

In comparing SLMs and MLMs, our experimental results clearly demonstrate that applying multilevel learning significantly improves the performance of the AttNet (3), AttNet (3 + 1), and EfficientNet-B0 models. However, it decreases the performance of the AttNet (1), AttNet (1 + 3), and ResNet18 models.

In the following subsection, we utilize all of the aforementioned models as the backbone of our proposed ingredient segmentation framework for multi-ingredient segmentation and assess their performance. This is because the performance of single-ingredient classification does not clearly correlate with the performance of multi-ingredient segmentation.

### 7.3. Evaluation on Ingredient Segmentation

As our objective is to identify multiple ingredients through multiple ingredient segments in food images, we evaluate the effectiveness of ingredient segmentation using metrics such as mIoU, mDice, mPurity, mEntirety, and mLoGTs, which are relevant to ingredient classification.

We evaluate the performance of ingredient segmentation on the FoodSeg103 dataset [15], which consists of images containing multiple ingredients along with pixel-level ingredient labels. However, the majority of food images in this dataset also include non-food background areas. Since our proposed method aims to segment only the ingredients in food images while excluding the background, the presence of the background might potentially affect the ingredient segmentation results. To address this issue, we replaced the background areas of these images with a blue background. In this process, each pixel in the background area is assigned an RGB value of (0, 0, 255), as this blue color is rarely encountered in food ingredients.

In this experiment, we employ mIoU, mDice, mPurity, mEntirety, and mLoGTs as evaluation metrics to assess the segmentation performance on this dataset. We also compare our mIoU with the one reported in [15]. However, it should be noted that the FoodSeg103 dataset comprises 103 ingredients, which is not entirely consistent with our defined 110 ingredient categories. As a result, direct comparison of the accuracy results presented in [15] may not be feasible.

#### 7.3.1. Analysis of Method 1

We discuss the segmentation performance of various backbones using Method 1, and the results are listed in Table 3. Based on the Purity results, SLM-EfficientNet-B0 achieves a highest score of 0.7679, followed by MLM-EfficientNet-B0 with a score of 0.694. However, these models exhibit low performance in terms of the Entirety score. Since our segmentation results will be used for the subsequent ingredient classification task, we consider these models not to be suitable for the segmentation used as backbones.

Next, we investigate the results of the metric Entirety. The MLM-AttNet models achieve relatively high values of over 0.8. As for LoGTs, we observe that MLM-AttNet (1) and MLM-AttNet (1 + 3) achieve comparatively good results of less than 0.1. Regarding IoU, the SLM-AttNet models achieve relatively high values of over 0.6. Finally, for Dice, both SLM-AttNet models and MLM-AttNet models have values of more than 0.7.

Because both the accurate segmentation of ingredients and the preservation of their integrity are crucial for the subsequent recognition process, we consider that under Method 1, MLM-AttNet (1) and MLM-AttNet (1 + 3) are suitable backbones for ingredient segmentation.

#### 7.3.2. Analysis of Method2

Here, we evaluate the performance of various models for ingredient segmentation using Method 2. The results are presented in Table 4.

It is important to note that the values of LoGTs are not equal to zero, despite the expectation that they should be zero according to the mechanism of Method 2. This discrepancy arises because we replaced the background region pixels of the images in the FoodSeg103 dataset with pure blue prior to ingredient segmentation. Consequently, when applying k-means clustering for pixel clustering, we set the number of clusters to K + 1, where K represents the number of ingredients in the image. This results in obtaining K + 1 segments, including K ingredient segments and one background segment. However, in some cases, we observed that certain areas of ingredients were mistakenly segmented into the background segment. Therefore, the LoGTs are not equal to 0, as the feature vectors of pixels corresponding to some ingredients and those of pixels corresponding to the background are clustered into the same cluster using the K-means algorithm.

From Table 4, we observe that the AttNet models outperformed the modified ResNet18 and modified EfficientNet-based models across all metrics. We further notice that various AttNet models under Method 2 exhibited superior performance compared to Method 1 in terms of Purity, LoGTs, IoU, and Dice metrics. Particularly, for Purity, the mPurity values of AttNet models under Method 2 improved by approximately 15% compared to those under Method 1. For IoU, the mIoU values showed an improvement of about 5%. In terms of Entirety, Method 2 achieved the highest value of 0.80, which was lower than the highest value of Method 1. In terms of LoGTs, SLM-AttNet (1) attained a highest value of 0.055. As for IoU, which is a comprehensive metric for segmentation evaluation, SLM-AttNet (1) achieved a score of 0.6532, while MLM-AttNet (1) achieved a score of 0.6540. Both scores are nearly identical.

Consequently, we believe that SLM-AttNet (1) under Method 2 serves as an optimal backbone for ingredient segmentation in terms of ingredient recognition.

On the other hand, the model size of SLM-AttNet (1) requires 22.113 MB of memory, whereas modified EfficientNet-B0 requires 70.506 MB. Moreover, as the backbone used in the ingredient segmentation framework, SLM-AttNet (1) requires less execution time than EfficientNet-B0 does. For instance, when taking an image of size 1024 × 1365 as input and obtaining all ingredient segmentation using Method 2, SLM-AttNet (1) takes 1.71 s to execute, while EfficientNet-B0 takes 2.04 s.

#### 7.3.3. Comparison with Previous Work

In comparison to previous work [15] of multi-ingredient segmentation, which achieves a class-wise mIoU of 0.439, our results demonstrate superior performance. Specifically, our MLM-AttNet (1) achieves the highest class-agnostic mIoU of 0.654 with Method 2, while the SLM-AttNet (1) achieves almost the same result of 0.6532 with Method 2. This indicates that our approach surpasses the previous work to some extent. Most importantly, our segmentation network uses a single-ingredient classification model as the backbone to generate masks for segmentation. This implies that we only need to train the ingredient classification model on a single-ingredient image dataset with image-level labels, thereby avoiding the need for pixel-level annotations.

#### 7.3.4. Visualization of Segmentation Results

To further investigate the performance of the ingredient segmentation framework, we present some examples of segmentation with different backbones. We compare three backbones: SLM-AttNet (1), MLM-AttNet (1), and EfficientNet-B0, and compare the results using Methods 1 and 2. We chose to compare the AttNet model with the EfficientNet-based model because it performed better than ResNet18 on the entity and LoGTs metrics.

**Figure 11:**
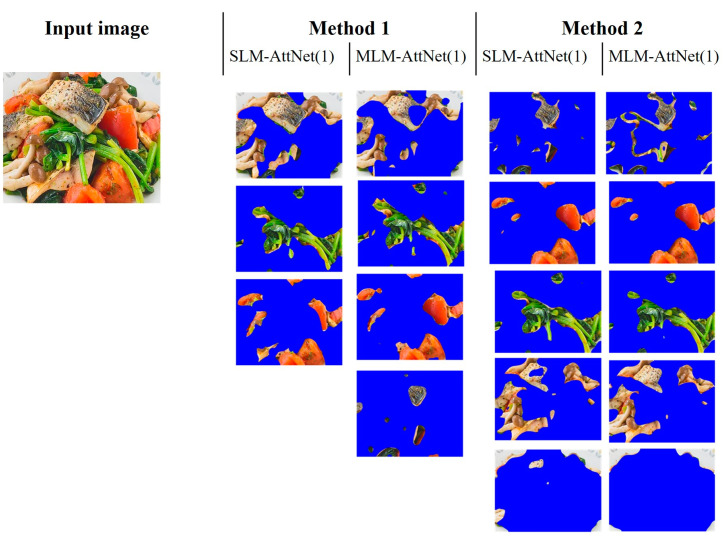
Segmentation results of four ingredients in a food image.

**Figure 12:**
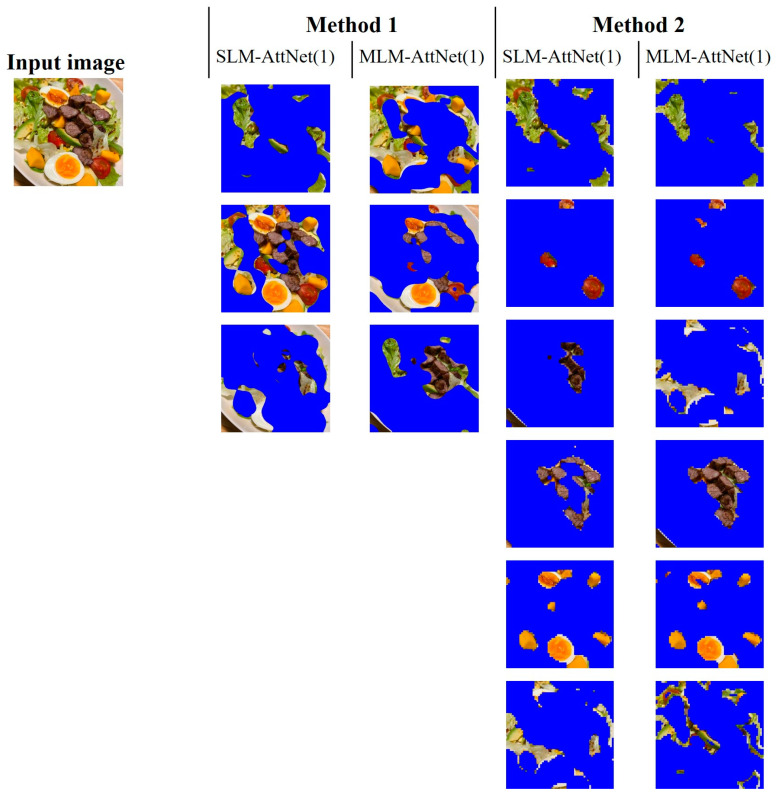
Segmentation results of six ingredients in a food image.

Regarding the comparison between Method 1 and Method 2, we conducted an analysis of two food images where the ingredients are highly mixed. As shown in Figure 11 and Figure 12, it can be observed that Method 1 is more effective at preserving the entirety of the ingredients, but struggles with the issue of mixing multiple types of ingredients together. In contrast, Method 2 excels in achieving higher segmentation purity, but is prone to splitting the same ingredient (e.g., fish flesh and fish skin in Figure 11) due to color differences, or merging different ingredients that have similar colors (e.g., egg yolk and mango in Figure 12). Overall, Method 1 is less effective for segmenting highly mixed ingredients, whereas Method 2 performs better in resolving this issue.

**Figure 13:**
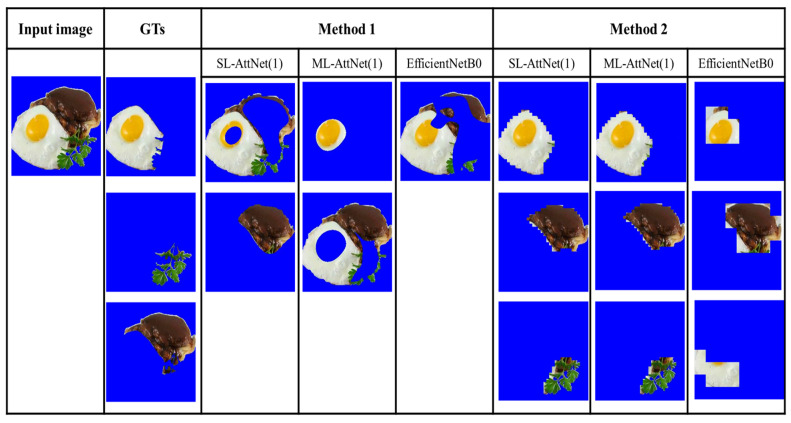
Three ingredients (egg, parsley, and steak) in a dish image from FoodSeg103 dataset.

**Figure 14:**
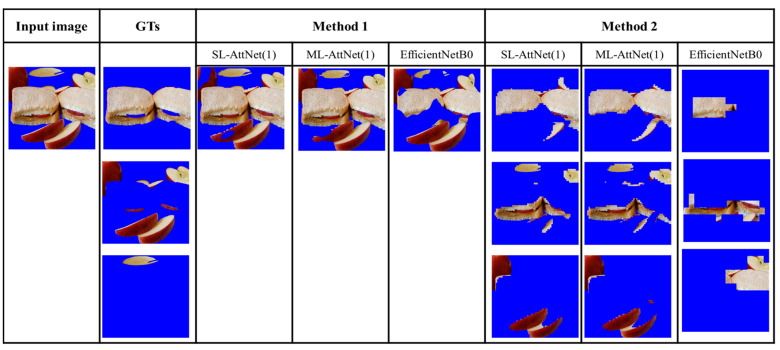
Three ingredients (bread, apple, and sauce) in a dish image from FoodSeg103 dataset. These results show that the proposed method has difficulty in segmenting ingredients having similar colors.

Regarding the comparison between AttNet (1) and EfficientNet-B0, we compare the results from SLM-AttNet (1) and SLM-EfficientNet-B0. As shown in Figure 13 and Figure 14, we found that the ingredient segmentation results generated by the AttNet (1) reserve more detailed boundaries than EfficientNet-B0. Furthermore, we observed that the segmentation results obtained by EfficientNet-B0 are reduced in quality through missing parts of the ingredients. In Figure 13, we clearly observed this issue, where EfficientNet-B0 completely misses the parsley region. In Figure 14, under Method 1, EfficientNet-B0 partially misses the sauce region; furthermore, under Method 2, EfficientNet-B0 completely misses the apple region.

Moving on to the analysis of segmentation results from the FoodSeg103 dataset, Figure 13 demonstrates that under Method 2, the egg is successfully segmented as a whole within the same segment. However, when employing Method 1, the segmentation of the egg is either separated or partially missed.

**Figure 15:**
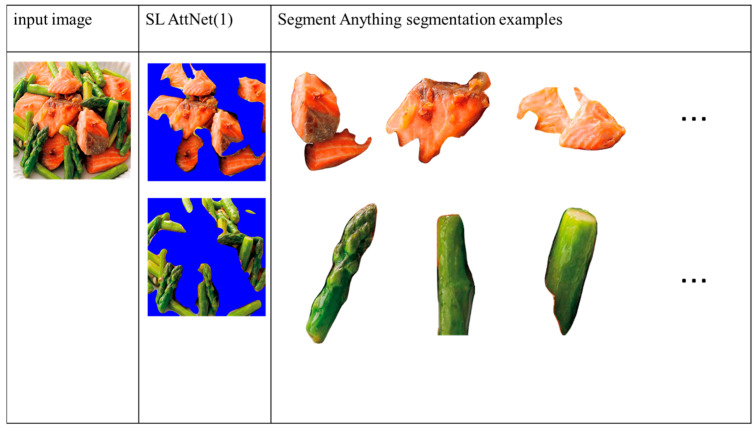
Comparison of segmentation results of our method with Segment Anything. Results show that the Segment Anything model over-segments the ingredient into small pieces that are difficult to identify.

**Figure 16:**
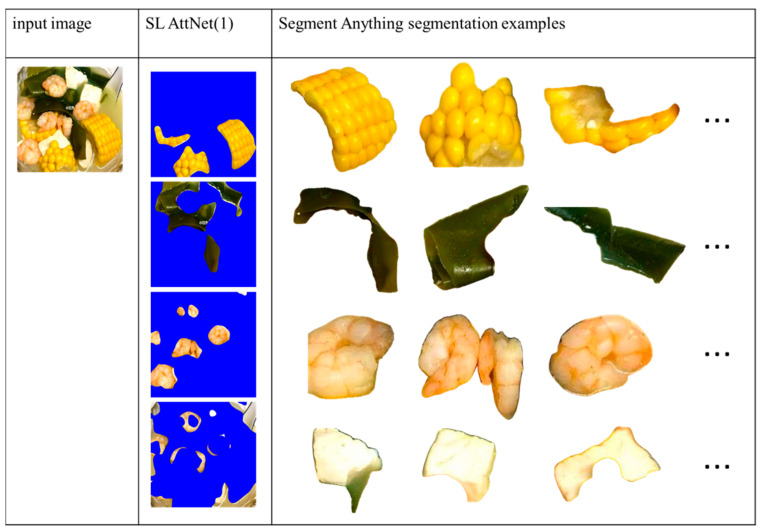
Comparison of the segmentation results of our method with Segment Anything. Results show that the Segment Anything model over-segments the ingredient into small pieces that are difficult to identify.

Finally, we compare the segmentation results of Method 2 with those of Segment Anything [25]. For our method, the SLM-AttNet (1) model is used for comparison. For Segment Anything, we use their published web demo to obtain the segmentation results via selecting the “segment everything” mode without adding any extra prompts. The segmentation results of the two multi-ingredient image examples are shown in Figure 15 and Figure 16, respectively. We observed that both models segmented the ingredients well, and Segment Anything returned more accurate boundaries than did the proposed method. However, Segment Anything over-segmented the ingredients into several small pieces that are difficult to distinguish. In contrast, our method grouped the same ingredient into the same segment. As our objective is to identify the ingredients in the food images, it is necessary to segment them as completely as possible. Therefore, we argue that the segmentation results obtained using our method are more suitable in terms of ingredient recognition.

### 7.4. Evaluation of Food Segmentation

In order to validate the generality of the proposed segmentation framework, the examination for food segmentation is introduced using the UECFoodPix COMPLETE dataset. Food segmentation generally refers to the process of segmenting food items from the background in an image. We select SLM-AttNet (1) as the backbone and use Method 2 for food segmentation. We calculate the mIoU and compare it with the results of previous studies, such as UEC FoodPix [29], GourmetNet [22], and BayesianGourmetNet [45]. Table 5 presents the results. Our ingredient segmentation framework performs better than UEC FoodPix but slightly worse than Deeplabv3+.

These results suggest that AttNet (1), a single-ingredient classification model, can be used as a backbone for partially addressing food segmentation. However, the proposed method is significantly inferior to GourmetNet. It appears that the single-ingredient classification model lacks the ability to segment dishes in the food images based on the proposed segmentation framework. Therefore, we plan to further explore the food classification model to enhance the performance of dish segmentation in the food images.

In summary, our qualitative and quantitative analyses indicate that the framework using SLM-AttNet (1) as the backbone and applying Method 2 to ingredient segmentation leads to better ingredient segments for the following ingredient recognition. However, we identify the drawbacks of the proposed approach: (1) some different ingredients, or some parts of the ingredients and the background in the image, may be segmented into the same segment and (2) some parts of the background may be segmented into the ingredient segments because of their similar visual features.

## 8. Conclusions

In this study, we introduced a hierarchical ingredient structure based on a standardized definition of ingredient categories for addressing the issue of the lack of ingredient datasets. This dataset selected 110 ingredient categories covering an entire food taxonomy, and collected 10,750 individual ingredient images with various cutting and cooking methods.

Then, we trained or fine-tuned the CNN-based single-ingredient classification models on the above dataset, and proposed a multiple ingredient segmentation framework that utilizes a single-ingredient classification model as the backbone to extract feature maps and generate masks for ingredient segmentation. This framework does not need pixel-level annotations, providing a more practical and cost-effective solution for food ingredient segmentation.

As a crucial component of this framework, we investigated the effectiveness of different backbone models for segmentation tasks. The significance of our experiments demonstrates that our proposed method, although simple, is highly effective. Importantly, our segmentation approach differs from methods relying on pixel-level annotation datasets, as we only need to train a single-ingredient classification model, eliminating the need for time-consuming and labor-intensive annotation datasets. We believe this is an important aspect for future research.

Finally, we evaluated the segmentation performance using five metrics—IoU, Dice, Purity, Entirety, and LoGTs—to explore the optimal backbone model and feature processing method. Our findings indicated that the optimal result was achieved when employing SLM-AttNet (1) and applying Method 2 for the ingredient segmentation.

Our proposed multi-ingredient segmentation framework lays the foundation and provides support for further advances in ingredient recognition, nutritional assessment, and recipe recommendations. Specifically, the accurate segmentation of ingredients enables more precise ingredient recognition, which in turn facilitates more accurate nutritional assessment, and more personalized recipe recommendations.

In future work, our objective is to tackle the issues of different ingredients being segmented into the same segment and some parts of the background being segmented into ingredient segments. We plan to enhance our ingredient segmentation framework through integrating a Food Segmentation module to remove non-food regions from images, thereby minimizing the influence of the background on ingredient segmentation.

Moreover, it is necessary to provide more results-based segmentation models to assess the effectiveness of the proposed method.

## Figures and Tables

**Table 1:** Architecture of the proposed AttNet network.

Stage	Operator	Resolution	Channels
1	Input	224 × 224	3
2	CB, k×k conv, stride 2	112 × 112	64
3	CB, k×k conv, stride 2	56 × 56	128
4	CB, k×k conv, stride 2	28 × 28	128
5	CB, k×k conv, stride 1	28 × 28	256
6	CB, k×k conv, stride 1	28 × 28	256
7	CB, k×k conv, stride 1	28 × 28	512
8	CB, k×k conv, stride 1	28 × 28	512
9	CB, j×j conv, stride 1	28 × 28	C#
10	avgpool, softmax	1 × 1	C#

**Table 2:** Performance of SLMs and MLMs on SI110 dataset for single-ingredient classification.

Types	Model	Accuracy	mPrecision	mRecall	mF1
SLM	AttNet (1)	0.2712	0.1933	0.1955	0.1693
	AttNet (1 + 3)	0.2716	0.1840	0.1946	0.1619
	AttNet (3 + 1)	0.2665	0.1846	0.1947	0.1623
	AttNet (3)	0.2553	0.1786	0.1805	0.1575
	EfficientNet-B0	0.8437	0.8161	0.8063	0.8017
	ResNet18	0.8684	0.8498	0.8571	0.8466
MLM	AttNet (1)	0.2326	0.1576	0.1544	0.1333
	AttNet (1 + 3)	0.2116	0.1787	0.1412	0.1285
	AttNet (3 + 1)	0.387	0.41	0.3254	0.324
	AttNet (3)	0.3205	0.3307	0.2686	0.2574
	EfficientNet-B0	0.8307	0.8290	0.8253	0.8168
	ResNet18	0.6940	0.6923	0.6603	0.6495

**Table 3:** Performance of multiple ingredient segmentation using SLMs and MLMs on FoodSeg103 with Method 1.

Types	Model	mPurity	mEntirety	mLoGTs	mIoU	mDice
SLM	AttNet (1)	0.6712	0.7555	0.1751	0.6051	0.7376
	AttNet (1 + 3)	0.6845	0.7394	0.1874	0.6028	0.7354
	AttNet (3 + 1)	0.6838	0.7675	0.1621	0.6056	0.7371
	AttNet (3)	0.6831	0.7791	0.1525	0.6072	0.7384
	EfficientNet-B0	0.7679	0.3116	0.6592	0.3666	0.5141
	ResNet18	0.5828	0.7879	0.1925	0.531	0.6789
MLM	AttNet (1)	0.6144	0.8463	0.0959	0.59	0.7265
	AttNet (1 + 3)	0.6036	0.8579	0.0917	0.5759	0.7157
	AttNet (3 + 1)	0.6225	0.7997	0.1410	0.5832	0.7213
	AttNet (3)	0.6167	0.8409	0.1087	0.5742	0.7137
	EfficientNet-B0	0.694	0.395	0.5804	0.4043	0.5597
	ResNet18	0.6594	0.4960	0.4805	0.434	0.5894

**Table 4:** Performance of multiple ingredient segmentation using SLMs and MLMs on Foodseg103 with Method 2.

Types	Model	mPurity	mEntirety	mLoGTs	mIoU	mDice
SLM	AttNet (1)	0.8339	0.8003	0.0552	0.6532	0.7665
	AttNet (1 + 3)	0.8565	0.7391	0.1276	0.6185	0.7407
	AttNet (3 + 1)	0.8255	0.6618	0.1824	0.5548	0.6911
	AttNet (3)	0.8158	0.6599	0.2072	0.5749	0.7024
	EfficientNet-B0	0.7922	0.6094	0.2657	0.5392	0.6780
	ResNet18	0.7980	0.1534	0.828	0.1327	0.2121
MLM	AttNet (1)	0.8256	0.7900	0.0837	0.6540	0.7611
	AttNet (1 + 3)	0.8373	0.7495	0.1141	0.6199	0.7415
	AttNet (3 + 1)	0.7949	0.5865	0.2487	0.5001	0.6473
	AttNet (3)	0.8026	0.6055	0.2336	0.5090	0.6533
	EfficientNet-B0	0.7559	0.5342	0.2984	0.3759	0. 5160
	ResNet18	0.7314	0.5963	0.2364	0.3645	0.4965

**Table 5:** Comparison of food segmentation performance with SOTA methods on UECFoodPix COMPLETE dataset.

Models	mIOU
UECFoodPix [29]	55.55%
GourmetNet [22]	65.13%
BayesianGourmetNet [45]	66%
Ours	60.18%

## Data Availability

Not applicable.

## Appendix A

List of 110 level 4 ingredient categories.

**Index**	**Ingredient Category**	**Index**	**Ingredient Category**	**Index**	**Ingredient Category**	**Index**	**Ingredient Category**
C1	green caviar	C29	yam	C57	daikon	C85	other white flesh
C2	raspberries	C30	apple	C58	grape	C86	sweat potato
C3	peach	C31	green onion	C59	pineapple	C87	broccoli
C4	enoki	C32	orange	C60	asparagus	C88	broad beans
C5	avocado	C33	apricot	C61	cheese	C89	Chinese chives
C6	konpu	C34	wakame	C62	cabbage	C90	bamboo shoot
C7	sesame seeds	C35	crab	C63	cream	C91	celery stem
C8	eel	C36	corn	C64	octopus	C92	lotos
C9	yogurt	C37	green soybean	C65	peas	C93	peanuts
C10	papaya	C38	hazel nuts	C66	fig	C94	chickpea
C11	bonito	C39	Bok choy	C67	kidney beans	C95	potato
C12	pitaya	C40	oyster	C68	sunflower seed	C96	pumpkin seeds
C13	chestnuts	C41	cauliflower	C69	lemon	C97	tomato
C14	pear	C42	cherry	C70	kiwi	C98	cattle
C15	purple laver	C43	lobster	C71	eggplant	C99	poultry
C16	salmon	C44	wax gourd	C72	grape fruits	C100	soybean
C17	banana	C45	almond	C73	mushroom	C101	onion
C18	Snow pea	C46	blueberry	C74	celtuce	C102	oyster mushroom
C19	black rice	C47	lettuce	C75	meat product	C103	cucumber
C20	bean sprout	C48	tree ears	C76	abalone	C104	pepper
C21	walnuts	C49	mackerels	C77	watermelon	C105	carrot
C22	tuna	C50	shimeji	C78	kidney bean	C106	shiitake
C23	melon	C51	pumpkin	C79	okra	C107	strawberry
C24	bitter melon	C52	cashews	C80	Chinese cabbage	C108	swine
C25	pistachio	C53	squids	C81	clam	C109	shrimp
C26	mantis shrimp	C54	mango	C82	egg	C110	wheat_product
C27	garlic stem	C55	millet	C83	pecan		
C28	rice	C56	spinach	C84	tofu		

## References

[1] Min W., Jiang S., Liu L., Rui Y., Jain R.C. (2018). A Survey on Food Computing. ACM Comput. Surv. (CSUR).

[2] Kagaya H., Aizawa K., Ogawa M. Food Detection and Recognition using Convolutional Neural Network. Proceedings of the 22nd ACM international conference on Multimedia.

[3] Aguilar E., Bolaños M., Radeva P. (2017). Food recognition using fusion of classifiers based on CNNs. Image Analysis and Processing-ICIAP 2017: 19th International Conference, Catania, Italy, 11–15 September 2017.

[4] Subhi M.A., Ali S.H., Mohammed M.A. (2019). Vision-Based Approaches for Automatic Food Recognition and Dietary Assessment: A Survey. IEEE Access.

[5] Lo F.P., Sun Y., Qiu J., Lo B.P. (2020). Image-Based Food Classification and Volume Estimation for Dietary Assessment: A Review. IEEE J. Biomed. Health Inform..

[6] Martinel N., Foresti G.L., Micheloni C. Wide-slice residual networks for food recognition. Proceedings of the 2018 IEEE Winter Conference on Applications of Computer Vision (WACV).

[7] Zhou F., Lin Y. Fine-grained image classification by exploring bipartite-graph labels. Proceedings of the IEEE Conference on Computer Vision and Pattern Recognition.

[8] Min W., Liu L., Luo Z., Jiang S. Ingredient-guided cascaded multi-attention network for food recognition. Proceedings of the 27th ACM International Conference on Multimedia.

[9] Qiu J., Lo F.P., Sun Y., Wang S., Lo B.P. Mining Discriminative Food Regions for Accurate Food Recognition. Proceedings of the British Machine Vision Conference.

[10] Bolaños M., Ferrà A., Radeva P. (2017). Food Ingredients Recognition Through Multi-label Learning. arXiv.

[11] Gao J., Chen J., Fu H., Jiang Y. (2022). Dynamic Mixup for Multi-Label Long-Tailed Food Ingredient Recognition. IEEE Trans. Multimed..

[12] Chen J., Zhu B., Ngo C., Chua T., Jiang Y. (2020). A Study of Multi-task and Region-Wise Deep Learning for Food Ingredient Recognition. IEEE Trans. Image Process..

[13] Xue Y., Niu K., He Z. Region-Level Attention Network for Food and Ingredient Joint Recognition. Proceedings of the 2021 4th International Conference on Algorithms, Computing and Artificial Intelligence.

[14] Chen J., Pan L., Wei Z., Wang X., Ngo C., Chua T. Zero-Shot Ingredient Recognition by Multi-Relational Graph Convolutional Network. Proceedings of the AAAI Conference on Artificial Intelligence.

[15] Wu X., Fu X., Liu Y., Lim E., Hoi S.C., Sun Q. A Large-Scale Benchmark for Food Image Segmentation. Proceedings of the 29th ACM International Conference on Multimedia.

[16] Wang Q., Dong X., Wang R., Sun H. Swin Transformer Based Pyramid Pooling Network for Food Segmentation. Proceedings of the 2022 IEEE 2nd International Conference on Software Engineering and Artificial Intelligence (SEAI).

[17] Xia X., Liu W., Wang L., Sun J. (2023). HSIFoodIngr-64: A Dataset for Hyperspectral Food-Related Studies and a Benchmark Method on Food Ingredient Retrieval. IEEE Access.

[18] Romero-Tapiador S., Tolosana R., Morales A., Espinosa-Salinas I., Freixer G., Fierrez J., Vera-Rodríguez R., Ortega-Garcia J., Pau E.C., Molina A.R. (2022). AI4Food-NutritionDB: Food Image Database, Nutrition Taxonomy, and Recognition Benchmark. arXiv.

[19] 生鮮食品品質表示基準 (Standards for Fresh Food Quality Labeling). https://www.caa.go.jp/policies/policy/food_labeling/quality/quality_labelling_standard/pdf/kijun_01.pdf.

[20] (2021). 新食品成分表 FOODS 2021, 新食品成分表編集委員会 (New Food Ingredients List FOODS 2021).

[21] Aguilar E., Remeseiro B., Bolaños M., Radeva P. (2017). Grab, Pay, and Eat: Semantic Food Detection for Smart Restaurants. IEEE Trans. Multimed..

[22] Sharma U., Artacho B., Savakis A. (2021). Gourmetnet: Food segmentation using multi-scale waterfall features with spatial and channel attention. Sensors.

[23] Okamoto K., Adachi K., Yanai K. Region-Based Food Calorie Estimation for Multiple-Dish Meals. Proceedings of the 13th International Workshop on Multimedia for Cooking and Eating Activities.

[24] Liang Y., Li J., Zhao Q., Rao W., Zhang C., Wang C. Image Segmentation and Recognition for Multi-Class Chinese Food. Proceedings of the 2022 IEEE International Conference on Image Processing (ICIP).

[25] Kirillov A., Mintun E., Ravi N., Mao H., Rolland C., Gustafson L., Girshick R. (2023). Segment Anything. arXiv.

[26] Chen J., Ngo C. Deep-based Ingredient Recognition for Cooking Recipe Retrieval. Proceedings of the 24th ACM international conference on Multimedia.

[27] Min W., Liu L., Wang Z., Luo Z., Wei X., Wei X., Jiang S. Isia food-500: A dataset for large-scale food recognition via stacked global-local attention network. Proceedings of the 28th ACM International Conference on Multimedia.

[28] Myers A., Johnston N., Rathod V., Korattikara A., Gorban A., Silberman N., Guadarrama S., Papandreou G., Huang J., Murphy K. Im2Calories: Towards an Automated Mobile Vision Food Diary. Proceedings of the 2015 IEEE International Conference on Computer Vision (ICCV).

[29] Okamoto K., Yanai K. UEC-FoodPix Complete: A Large-Scale Food Image Segmentation Dataset. Proceedings of the ICPR Workshops.

[30] Zhang X., Lu Y., Zhang S. (2016). Multi-Task Learning for Food Identification and Analysis with Deep Convolutional Neural Networks. J. Comput. Sci. Technol..

[31] Crawshaw M. (2020). Multi-task learning with deep neural networks: A survey. arXiv.

[32] Liang H., Wen G., Hu Y., Luo M., Yang P., Xu Y. (2021). MVANet: Multi-Task Guided Multi-View Attention Network for Chinese Food Recognition. IEEE Trans. Multimed..

[33] Dai J., He K., Sun J. Instance-Aware Semantic Segmentation via Multi-task Network Cascades. Proceedings of the 2016 IEEE Conference on Computer Vision and Pattern Recognition (CVPR).

[34] Cipolla R., Gal Y., Kendall A. Multi-task Learning Using Uncertainty to Weigh Losses for Scene Geometry and Semantics. Proceedings of the 2018 IEEE/CVF Conference on Computer Vision and Pattern Recognition.

[35] Li X., Zhou Y., Zhou Y., Wang W. (2021). MMF: Multi-task multi-structure fusion for hierarchical image classification. International Conference on Artificial Neural Networks.

[36] Victor S., Thomas W., Sebastian R. A hierarchical multi-task approach for learning embeddings from semantic tasks. Proceedings of the AAAI Conference on Artificial Intelligence.

[37] Dhanachandra N., Manglem K., Chanu Y.J. (2015). Image segmentation using K-means clustering algorithm and subtractive clustering algorithm. Procedia Comput. Sci..

[38] Zheng X., Lei Q., Yao R., Gong Y., Yin Q. (2018). Image segmentation based on adaptive K-means algorithm. EURASIP J. Image Video Process..

[39] Caron M., Bojanowski P., Joulin A., Douze M. Deep Clustering for Unsupervised Learning of Visual Features. Proceedings of the European Conference on Computer Vision.

[40] Van Gansbeke W., Vandenhende S., Georgoulis S., Van Gool L. Unsupervised semantic segmentation by contrasting object mask proposals. Proceedings of the IEEE/CVF International Conference on Computer Vision.

[41] Zhu Z., Dai Y. CNN-based visible ingredient segmentation in food images for food ingredient recognition. Proceedings of the 2022 12th International Congress on Advanced Applied Informatics (IIAI-AAI).

[42] Tan M., Le Q. Efficientnet: Rethinking model scaling for convolutional neural networks. Proceedings of the International Conference on Machine Learning, PMLR.

[43] He K., Zhang X., Ren S., Sun J. Deep Residual Learning for Image Recognition. Proceedings of the 2016 IEEE Conference on Computer Vision and Pattern Recognition (CVPR).

[44] Wang Y., Liu C., Zhu F., Boushey C.J., Delp E.J. Efficient superpixel based segmentation for food image analysis. Proceedings of the 2016 IEEE International Conference on Image Processing (ICIP).

[45] Aguilar E., Nagarajan B., Remeseiro B., Radeva P. (2022). Bayesian deep learning for semantic segmentation of food images. Comput. Electr. Eng..

